# The fates of ^15^N-labeled fertilizer in a wheat–soil system as influenced by fertilization practice in a loamy soil

**DOI:** 10.1038/srep34754

**Published:** 2016-10-07

**Authors:** Zhaoming Chen, Huoyan Wang, Xiaowei Liu, Dianjun Lu, Jianmin Zhou

**Affiliations:** 1 State Key Laboratory of Soil and Sustainable Agriculture, Institute of Soil Science, Chinese Academy of Sciences, Nanjing 210018, China; 2 University of Chinese Academy of Sciences, Beijing 100049, China

## Abstract

Appropriate fertilization practice is crucial to achieve maximum wheat grain yield with minimum nitrogen (N) loss. A field ^15^N micro-plot experiment was conducted to determine the effects of application methods [split application (SA) and band application (BA)] and N rates (60, 150 and 240 kg ha^−1^) on the wheat grain yield, urea-^15^N fate and N efficiency in Jiangyan County, China. At high N rates, wheat grain yield was significantly higher for SA than BA treatment, but there was no difference at the lower N rates. Plant N derived from fertilizer was higher in SA than in BA treatment. The high N fertilizer application increased total N uptake by wheat derived from fertilizer, but wheat plant N derived from soil was not affected by the N rate. Fertilizer-N recovery in SA treatment was higher than in BA treatment. Residual N recovery in the 0–80 cm soil layer was 31–51%, which decreased with increasing N rate. The highest N loss was found for BA treatment at the N application of 240 kg ha^−1^. The one-time BA of N fertilizer, especially for higher N rates, led to reduced wheat grain yield and N efficiency, and increased the N loss.

Wheat (*Triticum aestivum* L.) is one of three major cereal crops in China after maize (*Zea mays* L.) and rice (*Oryza sativa* L.) and the total production was 126 million tonnes in 2014^1^. Nitrogen (N) is one of the most important nutrients for the yield and quality of wheat^2^,^3^,^4^. A high N rate of 250 kg ha^−1^ for winter wheat is applied in Jiangsu Province, while the average grain yield of wheat is about 4.5 Mg ha^−1 ^5,6. Excessive use and inappropriate application of N fertilizer have resulted in low N use efficiency (NUE) and high N loss^7^. Therefore, appropriate N fertilization method and N rate are crucial for improving wheat grain yield and NUE, and reducing the negative effects of fertilization on the environment^8^,^9^.

Split application (SA) of N fertilizer is commonly used for winter wheat by farmers in China^5^,^10^,^11^,^12^. Numerous studies have shown that SA can enhance N recovery, grain yield and quality of wheat, and reduce N loss^13^. SA can also reduce the risk of wheat lodging^14^. However, SA is more labor- and energy-intensive and is inconvenient compared to a single application of fertilizer^14^,^15^. With developing urbanization, manpower for agriculture is increasingly deficient in China, as young farmers are increasingly migrating to cities for better-paying jobs^16^. A one-time band  application (BA) of solid urea is less time consuming than SA^17^. The topdressing of N fertilizer is usually broadcast by hand; however, this surface application results in more N loss through ammonia emission compared to the direct placement of N fertilizer in the soil^18^. N fertilizer applied in bands below the soil surface may be an effective practice for increasing yield and the N recovery efficiency of crops^19^. N fertilizer banded in the seed row has a toxic effect on the seed and reduces the germination of wheat^20^,^21^. Side-band application of N fertilizer allows high rates of fertilizer to be applied at one time and decreases the ammonia toxicity on seed^20^. Machines with one-pass seeding and banding fertilizer systems have been widely used in the North China Plain^22^. Simultaneous seeding and fertilizing saves time and labor^23^. However, the effects of a single band application of N fertilizer on wheat yield and the fate of urea-N in the plant–soil system have not been well documented in southeast China.

The grain yield of wheat increases with increasing N application rates in fields of low soil fertility^24^. However, the increase in wheat grain yield is not linearly related to the increase in N application rates, and grain yield can even decrease with high rates of N fertilizer^25^. Furthermore, some researchers have found that the NUE of wheat decreases with increased N application rate^26^,^27^. The N application rate should meet but not exceed crop demand, which is crucial to achieve maximum yield with minimum N loss^28^.

The ^15^N tracer technique has been widely used to monitor the fate of N fertilizer in the plant–soil system^2^,^26^. The objectives of this study were to determine the effects of fertilization practice and rate of N fertilizer on wheat yield, fate of urea-^15^N and N efficiency in a sandy soil in southeast China. We hypothesized that one-time band application of N fertilizer would increase the grain yield and NUE of winter wheat, and decrease N loss.

## Results

### Yields and N uptake by winter wheat

The dry matter yields of winter wheat increased with increasing N application rate (*p* ≤ 0.001), but were not affected by the application method of N fertilizer (Table 1). The interaction of fertilization method and application rate of N fertilizer only significantly affected the grain yield of winter wheat (Table 1). The wheat grain yield, straw yield and aboveground biomass (3.8, 4.3 and 8.1 Mg ha^−1^, respectively) were all significantly lower in the control treatment (CK) compared to all fertilization treatments (Table 2). The grain yields were within the range of 5.1–6.3 Mg ha^−1^ in SA and 5.3–5.8 Mg ha^−1^ in BA treatments. The straw yields were highest (7.6 Mg ha^−1^) at N rate of 240 kg ha^−1^ (N 240) for both the SA and BA treatments. The straw yields were within the range of 6.4–7.6 Mg ha^−1^ in SA and 6.0–7.6 Mg ha^−1^ in BA treatments. The highest total aboveground biomass was 13.9 Mg ha^−1^ for SA treatment at N 240, which was significantly higher than the remaining treatments, except for BA treatment at N 240. Due to serious lodging in the late growing stage, the harvest index (HI) was lower in the BA treatment at N 240 (0.43) compared to the other treatments (0.45–0.47).

Fertilization practice did not significantly affect N content in wheat, while application rate of N fertilizer had significant effects on N content (Table 1). N uptake in grain by wheat was within the range of 102.1–137.4 kg ha^−1^ in fertilized treatments, which were significantly higher than that for CK (76.6 kg ha^−1^) (Table 3). The N uptake was 3–5 times higher in grain than that in straw (14.9–51.0 kg ha^−1^). The total N content was significantly lower in CK (91.5 kg ha^−1^) than in fertilized treatments. The total N uptakes were within the ranges of 125.8–188.4 kg ha^−1^ in SA and 130.3–159.0 kg ha^−1^ in BA treatments, respectively. The total N content was higher in SA than in BA at N 240. The N uptake by grain and wheat plant were both affected by the interaction of fertilization practice and N rate (Table 1). The highest N harvest index (NHI) was determined in treatment CK (0.84); NHI decreased with increasing N application rate regardless of application method.

### Plant N derived from fertilizer and soil

Both fertilization practice and N rate significantly affected the N uptake by wheat derived from urea-N and soil (Table 1). N uptake in grain derived from ^15^N labeled urea (Ndff) and from soil (Ndfs) was within the range of 15.2–39.3% and 60.7–84.8%, respectively (Table 4). The Ndff and Ndfs in the total wheat plant were within the ranges of 14.6–40.3% and 59.7–85.4%, respectively. The Ndff (%) in grain was lower than that in straw (12.0–43.3%).

The placement method and N application rate significantly affected Ndff in grain, straw and total wheat plant, but had little effects on Ndfs in wheat (Table 1). The fertilizer-N uptake in grain was significantly higher in SA (34.4–54.0 kg ha^−1^) than that in BA treatment (24.7–30.8 kg ha^−1^), except for when the N rate was 60 kg ha^−1^ (N 60; Table 5). The N uptake in straw derived from fertilizer was in the range of 3.1–22.1 kg ha^−1^, which accounted for 16–29% of total Ndff in wheat; and, correspondingly, 71–84% of total Ndff was found in grain. At higher N application rates (150 and 240 kg ha^−1^), the total plant N derived from fertilizer was higher in SA (46.5–76.1 kg ha^−1^) than in BA (33.6–43.4 kg ha^−1^), but there was no difference in total Ndff between SA and BA treatment with N rate of 60 kg ha^−1^. The Ndff in wheat increased with increasing N application rate. At the N rate of 150 kg ha^−1^ (N 150), the grain N derived from soil was higher in SA than in BA. The N uptake in grain by wheat (79.8–93.2 kg ha^−1^) derived from soil was about 3–4 times that in straw (19.3–29.0 kg ha^−1^). The plant N derived from soil was greater than that derived from fertilizer. These results indicate that soil N is relatively important for wheat. The total N uptake derived from soil was similar between SA and BA treatments.

### Distribution of residual ^15^N fertilizer in soil

After wheat harvest, the residual N in the 0–80 cm soil layers was 25.8–75.0 kg ha^−1^ in SA treatment, accounting for about 31–43% of applied N fertilizer; and for BA treatment was 30.3–67.2 kg ha^−1^, accounting for about 30–51% of applied N fertilizer (Tables 6 and 7). About 59–83% of the residual N remained in the 0–20 cm soil layers. In the 20–40 cm soil layers, the residual N was in the range of 3.2–15.3 kg ha^−1^ in SA and 1.4–11.1 kg ha^−1^ in BA treatments. The residual N in soil was significantly affected by N rate, and fertilization method significantly affected residual N in 20–40, 40–60 and 60–80 cm soil layer (Table 1). The urea-N in soil decreased sharply at higher N application rates (N 150 and N 240) in soil layers deeper than 40 cm. The residual N increased and more residual N moved into the deeper soil layers with increasing N application rates (Table 6).

### Fate of urea-N in plant–soil system

The placement method and application rate of N fertilizer significantly affected N recovery in grain and wheat plant (Table 1). At higher N application rates (N 150 and N 240), N recovery in grain and total recovery in wheat were significantly higher in SA (22.5–22.9 and 31.0–31.7%, respectively) than in BA treatments (12.8–16.5 and 18.0–22.4%, respectively), while there was no significant difference at N 60 (Table 7). The N recovery in grain decreased with increasing N application rate in the BA treatment, while was similar among the three N rates in the SA treatment. Fertilizer N recovery in straw was higher in the SA treatment (7.5–9.2%) than that in the BA treatment (5.1–5.9%). N recovery in straw (5.2–9.2%) was about 25–50% lower than that in grain. The highest N loss (54.0%) was in the BA treatment at N 240, which was significantly higher than for the other five treatments (17.8–37.0%). Unaccounted N loss was not significantly different between the lower N rates (N 60 and N 150). Urea-N loss increased with increasing N application rate (*p* < 0.001). Soil residual N was in the range of 31.3–43.0% of applied N fertilizer in SA and 30.0–50.6% of applied N fertilizer in BA treatment. Fertilizer-N recovery in soil was higher at the lowest N application rate (N 60) than at N 150 and N 240.

### N efficiency

The application method of N fertilizer had no effect on the N apparent recovery efficiency (NARE), N agronomic efficiency (NAE) and partial factor productivity (PFP), but N rate significantly affected the N efficiency (Table 1). The NARE was higher in SA than in BA at N 240, but it was similar between SA and BA treatment at N 60 and N 150 (Table 8). On average, the NRE (mean of 28%) in wheat was one-third lower than NARE (mean of 44%). The results indicated that N fertilization enhanced the soil N uptake by wheat and there was a positive added N interaction (ANI). The NAE and PFP were in the ranges of 8.1–25.2 and 23.8–88.2 kg kg^−1^, respectively, and both decreased with increasing N application rates (*p* < 0.001). In SA, the N real use efficiency (NRUE) was not affected by the N application rate, but NRUE decreased significantly with increasing N fertilization rate in BA treatment.

## Discussion

The grain yield and total aboveground biomass were not significantly affected by the N application method, but increased with increasing N application rate (*p* < 0.001). In our results, the grain and total wheat yields were in the ranges of 5.1–6.3 and 11.3–13.9 kg ha^−1^, respectively (Table 2). Rees *et al*.^29^ reported that the dry matter yield of winter wheat did not significantly differ between surface application and BA of N fertilizer in Guanzhong Plain, northwestern China. Hartmann *et al*.^17^ found that the grain yield of winter wheat with SA of urea did not significantly differ from that with one-time BA at regreening stage of wheat in a two-season experiment in the North China Plain. The grain and straw yield of wheat was similar between fall and spring BA of N fertilizer in Saskatchewan, Canada^30^. The grain yield of wheat increased from 2.0 to 3.5 Mg ha^−1^ with increasing N rates from 0 to 180 kg ha^−1^ on a clay loam soil in 1994^24^. With BA of N fertilizer, the grain yield of durum wheat increased by 15% with increasing N application rate from 40 to 140 kg ha^−1^ in Indian Head, Canada^30^. Our results showed that the grain yields of wheat were similar between N 150 and N 240 in BA treatment, and were significantly lower than in SA treatment with N 240. The reason for this was that high application rates of N fertilizer at sowing reduced the wheat yield due to lodging. High rate of N application accelerated the early growth rate of wheat and resulted in excessive growth, and enhanced the risk of lodging^31^,^32^,^33^,^34^.

N uptake by wheat was in the range of 126–188 and 130–159 kg ha^−1^ in SA and BA treatments, respectively (Table 3). The total N content at N 240 was significantly higher in SA than in BA, because the wheat grain yield was significantly higher in SA compared with BA (Table 2). Some researchers reported that the N uptake by wheat did not differ between SA and spring-BA using urea^17^. Our study showed that the plant N uptake by wheat increased with increasing N application rate (*p* < 0.001). Xue *et al*.^35^ conducted a three-year experiment in the Taihu lake region of southeast China, and found that the total N uptake by wheat was 132 and 149 kg ha^−1^ at N application rates of 180 and 240 kg ha^−1^, respectively. Jia *et al*.^26^ showed that the total N uptake by winter wheat increased from 198 to 238 kg ha^−1^ when the N rate was increased from 150 to 270 kg ha^−1^, in a high-yield wheat cropping system (8.0–9.7 Mg ha^−1^) in the North China Plain.

In the present study, the fertilization practice had significant effects on the fertilizer-N utilized by winter wheat (*p* < 0.001). The total N uptake by wheat derived from urea fertilizer was higher in SA (20.7–76.1 kg ha^−1^) than in BA treatment (19.0–43.4 kg ha^−1^), showing that SA of N fertilizer could enhance N uptake from N fertilizer, especially for higher N application rates. Large amounts of N fertilizer applied at sowing time likely increased fertilizer-N immobilized by soil microorganisms, and then led to poor synchrony between N supply and crop demand^34^,^36^. López-Bellido *et al*.^13^ observed that topdressing of N fertilizer increased the Ndff in wheat compared to application at sowing time. The Ndff in wheat increased with increasing N application rate (*p* < 0.001). This trend was also reported by Wang *et al*.^25^, who found that the N uptake by wheat derived from fertilizer-N doubled when the N application rate increased from 96 to 240 kg ha^−1^. The total wheat plant N derived from soil was in the range of 103–117 kg ha^−1^, with no significant differences between all six treatments in our study. Some researchers also found that the N application rate did not affect the plant N derived from soil^24^,^26^.

Some results from ^15^N-labeled experiments showed that fertilizer-N recovery in wheat and unaccounted N loss were in the range of 17–53 and 10–46% in China, respectively^11^,^25^,^29^,^37^. In the present study, the N recovery in wheat and the N loss were in the ranges of 18–35 and 18–54%, respectively. Our results showed that the ^15^N recovery in wheat was higher in SA than in BA treatment when higher N rates (N 150 and N 240) were applied, but there was no difference for N 60. This contrasts with the results of Rees *et al*.^29^, who found that, at N application rates of 75 and 150 kg ha^−1^, the recovery of fertilizer-N was higher in BA (34 and 38%) than in surface application of N fertilizer (26 and 24%) in northwest China, respectively. They also found that the unaccounted N loss in BA treatment did not differ from that in surface-applied treatment. This contrasting response was most likely due to differences in rainfall – annual mean precipitation in the present study was 30% higher than that for Rees’ *et al*.^29^. The surface-applied N fertilizer absorbed by plants is dependent on subsequent rainfall and it cannot move into the root zone and be utilized by plants when rainfall is limited^19^. The other reason was that large amounts of N fertilizer were likely taken up by soil microbes in the early growing stage of wheat due to low wheat competition, and so high levels of N fertilizer were in organic forms in the soil during the rapid growth stage of wheat^36^,^38^. High application rate of N fertilizer at the early growth stage of the crop would enhance the unaccounted N losses through N leaching^34^.

The NARE (24–65%) was higher than NRE (18–35%) in all fertilized treatments (Table 8). This result indicated that N fertilization enhanced the soil N uptake by wheat and so a positive ANI occurred^39^. The result is also possibly due to pool substitution between fertilizer-N and soil N via immobilization of ^15^N-labeled fertilizer^6^,^40^. Zhao *et al*.^6^ found that the NRE in wheat measured by ^15^N tracing method was about one-third lower than that measured by the difference method in the Taihu Lake region, southeast China.

After the wheat was harvested, 30–51% of the applied N fertilizer remained in the 0–80 cm soil profiles and 59–83% of soil residual N was retained in the 0–20 cm soil layers. More fertilizer-N moved to the 20–40 cm soil layer when N fertilizer was applied at higher rates (N150 and N240) compared to N60 (Table 6). Shi *et al*.^5^ observed that the soil residual ^15^N after wheat harvest accounted for about 30% of the applied urea-N at the N application rate of 210 kg ha^−1^ in Jiangsu Province of China. Other researchers reported that 65% of N fertilizer remained in the 0–80 cm soil layer in a wheat–wheat cropping system in arid Morocco^41^. These results were similar to those of the present study. In our experiment, the N application rate significantly affected the fertilizer-N recovery in soil and high N application rate reduced soil residual N (Table 7). A ^15^N micro-plot experiment conducted in the North China Plain by Jia *et al*.^26^, which showed that the N recovery in soil was in the range of 28–37%, which decreased when the N rate was increased from 150 to 270 kg ha^−1^.

## Conclusions

Under the field conditions of the present study, SA of N fertilizer was a better fertilization practice for winter wheat compared to one-time BA. At lower N application rates (N 60 and N 150), there was no difference in grain yield of winter wheat between SA and BA treatments; however, at N 240, the grain yield was significantly higher in SA than in BA treatment. Fertilizer-N recovery was higher in SA, especially at higher N rates (N 150 and N 240), than in BA treatment. High N application rates decreased the N recovery in soil and increased the N loss. Unaccounted N loss was the highest in BA treatment (54%) at N 240. The local Department of Agriculture’s recommended N rate is 225 kg ha^−1^ for winter wheat. Therefore one-time BA is not recommended for the loamy soil in this region. BA of N fertilizer coupled with SA may be effective for winter wheat with a seven-month growing season; however, this requires further investigation.

## Materials and Methods

### Site description

The field ^15^N micro-plot experiment was conducted at Jiangyan County of Jiangsu Province (32°31′N, 120°9′E) in southeast China, during 2014–2015. The region is classified as sub-tropical with a monsoon climate. The average annual rainfall is 991.7 mm and the average temperature is 14.5 °C. The soil is developed from the ancient Yellow River, and is classified as a Luvisols with a loam texture (IUSS working group WRB, 2014)^42^. The 0–20 cm soil properties were as follows: soil bulk density 0.90 g cm^−3^, total carbon 22.60 g kg^−1^, total N 1.64 g kg^−1^, Olsen-phosphorus (P) 16.63 mg kg^−1^, available potassium (K) 85.40 mg kg^−1^ and pH (soil: water, 2.5:1) 7.75, sand 44.7%, slit 46.4%, and clay 8.9%. Summer rice–winter wheat rotation was used in the experimental site. Rice was transplanted in early June and harvested in later October, and wheat was directly seeded in early November and harvested in early June in the region. The soil was plowed down to 10–15 cm by tillage rotary before planting rice and wheat. No irrigation was applied during the wheat growing season.

### Experiment design

The ^15^N micro-plots were established by plastic frames with area of 0.25 m^2^ (0.5 m × 0.5 m) and a height of 0.35 m to monitor the fates of ^15^N-labeled fertilizer. The plastic frames were pressed to 0.3 m deep into the soil and kept 0.05 m above the soil to prevent runoff and lateral contamination of unlabeled N fertilizer. The topsoil (0–30 cm) was dug out and mixed with basal fertilizers, and then backfilled the micro-plot. The wheat cultivar Yangmai16 was used, which showed good productivity and adaptability in Jiangyan County. Two rows of seeds were sown in each micro-plot at a seed rate of 180 kg ha^−1^ and with row spacing of 25 cm. The wheat was planted in early November 2014 and harvested in early June 2015. The experiment adopted a randomized block design with three replicates. The field trial included two application methods and three N application rates. The two application methods were SA, as used by local farmers, and one-time BA. The three N rates were 60, 150 and 240 kg ha^−1^ termed N 60, N 150 and N 240, respectively. In SA, ^15^N-labeled urea was broadcast by hand at sowing (24, 60 or 96 kg N ha^−1^), at tillering (18, 45 or 72 kg N ha^−1^) and at jointing (18, 45 or 72 kg N ha^−1^). In the BA treatment, the ^15^N-labeled urea (60, 150 or 240 kg N ha^−1^) was applied in a band at 5 cm from the seed and 10 cm under the soil surface. A blank control treatment (CK) was included in which no N fertilizer was applied. The wheat was planted around the micro-plot to reduce edge effects, and the application method and N rate were the same as the corresponding micro-plots. The ^15^N-labeled urea (^15^N abundance of 10.15%) was provided by the Shanghai Research Institute of Chemical Industry. The P fertilizer (150 kg P_2_O_5_ ha^−1^, triple superphosphate) and K fertilizer (180 kg K_2_O ha^−1^, potassium chloride) were applied to all treatments at sowing.

### Plant and soil sampling and ^15^N analyses

The wheat was harvested by hand in each micro-plot after wheat maturity and divided into grain and straw. The fresh grain and straw samples were dried at 70 °C to constant weight for determination of dry weight. The dry samples were ground into powder and passed through a 150-μm screen before total N and ^15^N analysis.

Soils were sampled to a depth of 80 cm in 20-cm increments. In SA treatment, the soil samples were randomly collected from four points in the micro-plots and segments of the same depth were mixed well as a single soil sample. Four soil cores for BA treatment were collected: two from the fertilizer band and the other two from the middle of two wheat rows. The same depth segments of four soil cores were mixed well as a single soil sample. The soil samples were air-dried and ground to pass through a 150-μm screen for total N and ^15^N analysis. Soil bulk density was determined after harvesting wheat using the cutting ring method. Grain, straw and soil samples were analyzed for total N and ^15^N abundance using an elemental analyzer (Costech ECS4010, Costech Analytical Technologies Inc., Valencia, CA, USA) coupled to an isotope ratio mass spectrometer (Delta V Advantage, Thermo Fisher Scientific Inc., Waltham, MA, USA).

### Calculation methods

All ^15^N was expressed as the atom percent (%) excess corrected for background abundance (0.366%).

The amounts of plant N derived from fertilizer (Ndff) and the soil residual N were calculated as follows^13^,^26^:
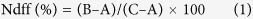
Where A is the ^15^N natural abundance, B is the ^15^N atom percent excess in the plant or soil and C is the ^15^N atom percent excess in the N fertilizer.
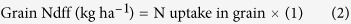

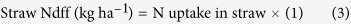

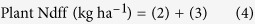

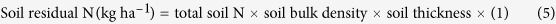


The N efficiency parameters were calculated as follows^13^,^25^,^43^:
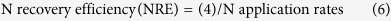

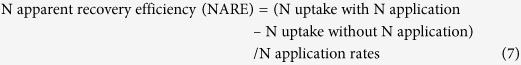

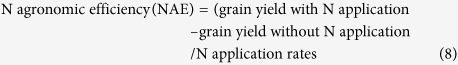

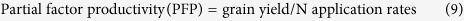

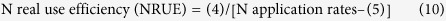
The NRUE can directly assess the impact of fertilizer management practices on environment because it only involves the fertilizer-N uptake by plant and loss, and residual N is not considered in NRUE because soil residual N can be absorbed by the succeeding crops^43^.

### Statistical analysis

Statistical analysis was conducted using SPSS 16.0 (SPSS Inc., Chicago, USA) for analysis of variance (ANOVA). Two-way ANOVA was used to assess the effects of fertilization practice and application rates of N fertilizer on wheat yield, N uptake, N efficiency and fates of urea-^15^N. Differences among treatments were compared by the least significance difference at the 5% level.

## Additional Information

**How to cite this article**: Chen, Z. *et al*. The fates of ^15^N-labeled fertilizer in a wheat–soil system as influenced by fertilization practice in a loamy soil. *Sci. Rep.* **6**, 34754; doi: 10.1038/srep34754 (2016).

## Figures and Tables

**Table 1:** Results of two-way ANOVA (*p* values) from the effects of fertilization practice, N rate and their interaction on dry matter yield, harvest index (HI), N content, N harvest index (NHI), percent of N derived from fertilizer and soil (%), amount of N derived from fertilizer and soil (kg ha^−1^), soil residual N, fertilizer N recovery in wheat and soil, N loss and N efficiency.

Effect	DF	Dry matter yield			HI	N content			NHI	Ndff (%)			Ndfs (%)			Ndff (kg ha^−1^)		
		Grain	Straw	Total		Grain	Straw	Total		Grain	Straw	Total	Grain	Straw	Total	Grain	Straw	Total
Practice (P)	1	0.187	0.294	0.179	0.886	0.183	0.185	0.117	0.485	<0.001	<0.001	<0.001	<0.001	<0.001	<0.001	<0.001	<0.001	<0.001
N rate (N)	2	<0.001	0.001	<0.001	0.112	<0.001	<0.001	<0.001	0.001	<0.001	<0.001	<0.001	<0.001	<0.001	<0.001	<0.001	<0.001	<0.001
P × N	2	0.023	0.715	0.815	0.036	0.018	0.236	0.029	0.586	0.002	0.003	0.001	0.002	0.003	0.001	<0.001	0.005	<0.001
		Ndff (kg ha^−1^)			Residual ^15^N in soil					N recovery			Soil Residual	Loss	N efficiency			
		Grain	Straw	Total	0–20 cm	20–40 cm	40–60 cm	60–80 cm	0–80 cm	Grain	Straw	Total			NARE	NAE	PFP	NURE
Practice (P)	1	0.032	0.599	0.051	0.170	0.032	0.052	0.006	0.582	0.001	<0.001	<0.001	0.272	0.120	0.711	0.931	0.931	0.029
N rate (N)	2	0.815	0.014	0.408	<0.001	<0.001	0.015	<0.001	<0.001	<0.001	0.331	0.003	0.001	<0.001	<0.001	<0.001	<0.001	<0.001
P × N	2	0.251	0.687	0.506	0.880	0.092	0.506	0.002	0.094	0.040	0.313	0.047	0.287	0.041	0.173	0.355	0.358	0.052

**Table 2:** Effects of fertilization practice and N rate on dry matter yield and harvest index (HI) of winter wheat.

Treatment		Dry matter yield (Mg ha^−1^)			HI
Practice	N rate	Grain	Straw	total	
SA	N 60	5.1c	6.4bc	11.5cd	0.45ab
	N 150	5.8b	7.0ab	12.8b	0.46ab
	N 240	6.3a	7.6a	13.9a	0.46ab
BA	N 60	5.3c	6.0c	11.3d	0.47a
	N 150	5.8b	6.6c	12.4bc	0.47a
	N 240	5.7b	7.6a	13.3ab	0.43b
CK		3.8d	4.3d	8.1e	0.47a

Different letters within a column represent significant differences (*p* < 0.05).

CK: N application 0 kg ha^−1^; SA: conventional split application; BA: band application.

**Table 3:** Effects of fertilization practice and N rate on N content in wheat and N harvest index (NHI) of winter wheat.

Treatment		N content (kg ha^−1^)			NHI
Practice	N rate	Grain	Straw	total	
SA	N 60	102.1c	23.7d	125.8c	0.81ab
	N 150	114.2bc	35.5bc	149.7b	0.76cd
	N 240	137.4a	51.0a	188.4a	0.73d
BA	N 60	104.6c	25.7cd	130.3c	0.80abc
	N 150	117.9b	32.2bcd	150.1b	0.79bc
	N 240	117.7b	41.3ab	159.0b	0.74d
CK		76.6d	14.9e	91.5d	0.84a

Different letters within a column represent significant differences (*p* < 0.05).

CK: N application 0 kg ha^−1^; SA: conventional split application; BA: band application.

**Table 4:** Effects of fertilization practice and N rate on N uptake by wheat derived from fertilizer (Ndff) and soil (Ndfs).

Treatment		Ndff (%)			Ndfs (%)		
Practice	N rate	Grain	Straw	Total	Grain	Straw	Total
SA	N 60	16.0d	19.0e	16.5e	84.0a	81.0b	83.5a
	N 150	30.1b	34.4b	31.1b	69.9c	65.6e	68.9d
	N 240	39.3a	43.3a	40.3a	60.7d	56.7f	59.7e
BA	N 60	15.2d	12.0f	14.6e	84.8a	88.0a	85.4a
	N 150	20.9c	27.7d	22.4d	79.1b	72.3c	77.6b
	N 240	26.1b	30.4c	27.2c	73.9c	69.6d	72.8c

Different letters within a column represent significant differences (*p* < 0.05).

SA: conventional split application; BA: band application.

**Table 5:** Effects of fertilization practice and N rate on N uptake by wheat derived from fertilizer (Ndff) and soil (Ndfs).

Treatment		Ndff (kg ha^−1^)			Ndfs (kg ha^−1^)		
Practice	N rate	Grain	Straw	Total	Grain	Straw	Total
SA	N 60	16.2d	4.5d	20.7d	85.9ab	19.3b	105.2
	N 150	34.4b	12.2b	46.5b	79.8b	23.3ab	103.2
	N 240	54.0a	22.1a	76.1a	83.4ab	29.0a	112.4
BA	N 60	15.9d	3.1d	19.0d	88.7ab	22.6ab	111.3
	N 150	24.7c	8.9c	33.6c	93.2a	23.3ab	116.5
	N 240	30.8bc	12.6b	43.4b	87.0ab	28.7a	115.7

Different letters within a column represent significant differences (*p* < 0.05).

SA: conventional split application; BA: band application.

**Table 6:** Effects of fertilization practice and N rate on the residual ^15^N in the soil profiles.

Treatment		Residual ^15^N (kg ha^−1^) in soil (cm)				
Practice	N rate	0–20	20–40	40–60	60–80	0–80
SA	N 60	19.8c	3.2c	0.8c	2.0b	25.8d
	N 150	36.5b	11.0b	2.8abc	2.5b	52.7c
	N 240	44.2ab	15.3a	3.0abc	12.5a	75.0a
BA	N 60	25.0c	1.4c	1.5bc	2.4b	30.3d
	N 150	38.6ab	11.1b	4.3ab	2.2b	61.0bc
	N 240	47.8a	9.3b	6.1a	3.9b	67.2ab

Different letters within a column represent significant differences (*p* < 0.05).

SA: conventional split application; BA: band application.

**Table 7:** Effects of fertilization practice and N rate on fate of urea-N in soil–plant system.

Treatment		N recovery (%)			Soil residual (%)	Loss (%)
Practice	N rate	Grain	Straw	Total		
SA	N 60	27.0a	7.5ab	34.5a	43.0ab	22.5cd
	N 150	22.9a	8.1a	31.0a	35.2bcd	33.8bc
	N 240	22.5a	9.2a	31.7a	31.3cd	37.0b
BA	N 60	26.5a	5.1c	31.6a	50.6a	17.8d
	N 150	16.5b	5.9bc	22.4b	40.7abc	37.0b
	N 240	12.8b	5.2c	18.0b	30.0d	54.0a

Different letters within a column represent significant differences (*p* < 0.05).

SA: conventional split application; BA: band application.

**Table 8:** Effects of fertilization practice and N rate on the NRE, NARE, NAE, PAP and NRUE.

Treatment		NRE (%)	NARE (%)	NAE (kg kg^−1^)	PFP (kg kg^−1^)	NRUE (%)
Practice	N rate					
SA	60	34.5a	57.2a	22.3a	85.2a	61.3ab
	150	31.0a	38.8b	13.7b	38.9b	47.9bc
	240	31.7a	40.4b	10.7c	26.4c	46.1bc
BA	60	31.6a	64.6a	25.2a	88.2a	65.1a
	150	22.4b	39.0b	13.7b	38.9b	37.9c
	240	18.0b	24.1c	8.1c	23.8c	25.2d

Different letters within a column represent significant differences (*p* < 0.05).

SA: conventional split application; BA: band application; NRE: N recovery efficiency; NARE: N apparent recovery efficiency; NAE: N agronomy efficiency; PFP: partial factor productivity; NRUE: N real use efficiency.
